# Screen Printed Particle-Based Microfluidics: Optimization and Exemplary Application for Heavy Metals Analysis

**DOI:** 10.3390/mi14071369

**Published:** 2023-07-04

**Authors:** Indrek Saar, Hanno Evard

**Affiliations:** Institute of Chemistry, University of Tartu, Ravila 14a, 50411 Tartu, Estonia; evardhanno@gmail.com

**Keywords:** microfluidics, µPADs, porous material microfluidics, screen printing, thin-layer chromatography, heavy metals

## Abstract

In this work, a screen-printing method was developed to create porous particle-based materials as layers with specifically designed shape to produce microfluidics systems. Among several tested binding agents, xanthan gum was found to be an excellent choice for a printing mixture thickener as well as a durable binder for the resulting material. In addition to demonstrating control over the shape of the printed microfluidics chips, control over material thickness, wetting characteristics and general method accuracy were also investigated. The applicability of the introduced method was further demonstrated with a development of an exemplary microfluidics chip for quantitative detection of Fe (III), Ni (II), Cu (II), Cd (II), and Pb (II) from a mixed sample at millimolar levels. The novel approaches demonstrated in this article offer new perspective into creating multiplexed on-site chemical analysis tests.

## 1. Introduction

The development of chemical sensors and analysis devices has been a major challenge addressed by microfluidics [1,2]. The concept of lab-on-a-chip is particularly fascinating, as it would enable the entire chemical analysis, from sample addition to result acquisition, to be performed on a single miniature chip [2,3,4]. Despite the extremely appealing nature of this approach, the technology itself has not yet found its way into wider use [5]. Among the different obstacles in commercializing the currently developed systems is the reliance on external pumps, power sources, and detecting devices, which significantly increase the complexity and overall cost [6]. Fortunately, the need for external devices can be greatly avoided in case of paper-based microfluidic devices (µPADs) [7,8], which use the already existing porous structure of paper for passive liquids transport by capillary action. In case of µPADs, the microfluidics systems are mostly achieved by cutting out a specific shape or blocking certain areas of the material [9]. However, most of the used µPAD production methods either lack resolution, do not allow mass production, or the equipment for prototyping is costly [10,11]. In addition to manipulating the geometry of the microfluidics system, control over material thickness, porosity, and surface chemistry is equally important in determining the performance of the device [12]. However, these characteristics rely largely on commercially available paper materials, which means that in most cases all of the desired properties (thickness, porosity, etc.) are not combined in a single paper or the chosen fabrication method might be unsuitable for that specific material.

A significant improvement would be to use particle-based materials, where the final properties of the formed material can be altered by simply choosing particles with the right size distribution and surface chemistry. Similar to µPADs, the pores between the particles form a network that allows capillary action. Furthermore, when combined with having control over the shape and layer thickness during the material fabrication process (from the chosen particles), the resulting method for producing porous microfluidics devices would be substantially more efficient and versatile. Although some work has demonstrated how to form precise shapes out of particle-based materials for microfluidics [13,14,15], there has been little development in this direction. Similar to those approaches, utilizing a 3D printer to create porous materials from particles has also been reported several times [16,17,18], but that is not suitable for large scale production. In the previous work published in the article by Evard et al. [19], several novel approaches were demonstrated to achieve that goal, of which screen printing (SP) appeared to be the most promising for further development. SP is a fast, simple, and relatively inexpensive production method that is also scalable for mass production. However, some issues in using SP in our previous work [19] remained (such as low resolution and undesired change in the printing mixture viscosity during printing), and further optimization was necessary.

Developing rapid tests for screening heavy metals can be considered among the most popular challenges addressed by microfluidics [10,20]. Metals such as nickel, cadmium, copper, lead, and iron are among the most common heavy metals present in the environment, while lead and cadmium could be considered among the most dangerous toxic metals [21,22]. Therefore, especially appealing is the development of multiplexed analysis tests that allow to detect several of these metals simultaneously, saving time and labour while also providing more representative results regarding each sample. Most of the µPADs that currently demonstrate this rely on a multi-armed approach where the same sample solution reaches several separate detection zones [23,24,25]. To achieve the necessary analytical performance, these approaches require pre-treatment zones, specific for each analyte, which usually contain several different masking reagents and control the pH, making the chip preparation time-consuming, while diminishing their mass-producibility. Furthermore, the risk of interference and diffusion of the reagents or products from the pre-treatment to detection zone remains [23,26]. A more fundamental alternative would be to use chromatography for the separation of analytes, significantly increasing the selectivity and also allowing the use of less selective yet highly sensitive reagents (e.g., dithizone) [27], making the overall production of the chip faster and less expensive.

In the current article, a variety of different binders are tested to solve the abovementioned issues with SP. Moreover, other printing parameters are studied to determine their influence and general optimal conditions for creating porous layered materials out of microparticles with SP were found. To demonstrate the applicability of the investigated approach, an exemplary microfluidics chip for separation and colorimetric detection of Ni (II), Cd (II), Cu (II), Pb (II), and Fe (III) is developed.

## 2. Materials and Methods

### 2.1. Used Chemicals

The different silica gel particles used in this study included LiChroprep Si 60 (15–25 µm) (Sigma-Aldrich, St. Louis, MO, USA), LiChrospher Si 60 (5 µm) (Sigma-Aldrich), Davisil grade 710 (10–14 µm) (Supelco), and Si 60 (63–200 µm) (Honeywell Fluka, Seelze, Germany) particles, which were further sieved to narrow the size distribution down to 63–142 µm. The tested binding agents are as follows: alginic acid sodium salt (Acros Organics, Geel, Belgium), guar gum (Sigma-Aldrich), xanthan gum (XG) from Xanthomanas camperstis (Sigma-Aldrich), XG (Piprapood (Tallinn, Estonia) local provider), agarose (Fischer Scientific, Hampton, NH, USA), and potato starch (from local supermarket polyethylene glycol (PEG35000, Sigma-Aldrich).

For metal cation analysis, iron (III), lead (II), cadmium (II), nickel (II), and copper (II) nitrates from Sigma-Aldrich were used. A total of 0.125 M sodium nitrate (Sigma-Aldrich) solution and 0.2 M sodium acetate buffer (pH 5) from sodium acetate and glacial acetic acid were prepared to use as eluents. For metal detection, 0.02% (*w*/*v*) and 0.04% (*w*/*v*) dithizone (Alfa Aesar) solution in acetone, 0.5% (*w*/*v*) dimethylglyoxime solution in ethanol, and 1% (*w*/*v*) aquas potassium ferrocyanide solution were used. In case of metal detection related experiments, deionized water (Millipore, Milli-Q Advantage A10, Burlington, MA, USA) was used. Unless stated otherwise, all chemicals used were of at least reagent grade.

### 2.2. Screen Printing Equipment

Three different screens with aluminium frames with T90, T48, and T14 polyester meshes (Seritek OÜ, Tallinn, Estonia) were used with mesh openings of 66 µm, 142 µm, and 514 µm, respectively. All screens had their meshes stretched at a 45 degrees angle to the frame. Unless specified otherwise, the screen with T48 mesh was then used for printing.

Cricut Joy (Cricut, Inc., South Jordan, UT, USA) cutter-plotter with Cricut Design Space software was used to design and cut out masks from removable vinyl (ORACAL 641), which were then applied on the screens to form the stencil. The designed mask for the experiments performed to investigate SP consisted of six separate shapes: two bigger rectangular areas connected with four narrow channels (Supplementary Figures S2 and S3). The designed widths of the channels varied from 0.1 mm to 2.4 mm in 0.1 mm steps. The channels allowed to determine the resolution of printing (the minimal achievable width for channels or elements), while the large areas showed how uniform the resulting material was.

In all cases, the chip (the material printed onto glass) was formed with the following glass slides: either 76 × 26 mm Microscope Slides (VWR) or, in case of metal analysis chip, 75 × 50 mm Micro Slides (Corning, Somerville, MA, USA).

### 2.3. Printing Mixtures and Technique

Unless stated otherwise, then for the SP investigation measurements, the slurry made for printing consisted of 470 mg silica gel particles (LiChroprep 15–25 µm) and 75 µL of glycerol per 1 millilitre of binding agent solution (made in distilled water with 4 mg mL^−1^ of binder). Glycerol was added to improve the flow and prevent the mixture from drying too fast on the screen, resulting in higher repeatability. The slurry was freshly made before each printing session and thoroughly mixed by hand into a homogenous mixture.

SP was conducted manually with the usual techniques. The distance between the glass slide and mesh (off-contact) was set to 2.2 mm. Flooding of the screen was done before every print and the first print was discarded in each session to achieve higher repeatability of the results. In case of investigation and characterization of SP, 6 chips were printed simultaneously, and 3 consecutive prints were done for each experiment series, resulting in total of 18 samples. After printing, the plates were dried in an oven at 90 °C overnight (for at least 12 h) to let all the water and glycerol evaporate. An illustration of the setup and main steps of the SP process can be found in Supplementary Figure S3.

In case of the chips developed for metal analysis, some changes in mixture as well as in the following treatment process were made. Firstly, alternative silica gel particles were used since LiChroprep silica gel provided an interfering signal with dithizone (see Supplementary Interference with dithizone for further information). Davisil grade 710 particles (10–14 µm) were found to be more suitable since they displayed less of that interference, and it was easier to overcome with material pre-treatment steps. Because of the slightly different properties of the particles, the new optimal mixture consisted of 370 mg of particles per 1 mL of XG solution (4 mg ml^−1^). Furthermore, glycerol was not included in the printing mixture as some residual amount of it interfered with the detection of analytes. As a result, less prints could be completed with the same amount of mixture in one printing session due to the mixture drying much faster. However, only 2 h of drying at 90 °C was sufficient because of the absence of glycerol.

### 2.4. Instrumentation for Characterization and Detection

To determine the flow characteristics of the material, approximately 1 mm of the bottom rectangular-shaped area of the material was submerged into an aqueous solution of xylenol orange (0.2%) and the time it took to wet the 2 cm long channel was measured. A millimetre paper was placed on the back of the glass slide and pictures were taken with a camera as the material wetted (Supplementary Figure S4). The length of the wetted material as a function of time was recorded, and the approximate wetting times were calculated (in units of s/2 cm). Whatman grade 1 (GE Healthcare, Chicago, IL, USA) filter paper was cut to a similar pattern as the printed materials with Cricut Joy to measure the wetting times in comparison with the printed materials using an aqueous solution of blue food colouring agent.

The thickness of the printed material was determined using a digital YATO YT-72305 micrometre with 0.002 mm accuracy, by determining the thickness of the substrate plate and then covering it with another glass slide and subtracting the plate thicknesses from the total.

The accuracy of the printing process (how closely the printing result resembles the design) was estimated by measuring the widths of the channels with Leica optical microscope (M165 FC) with 7.3× magnification and Leica Application Suite (version 3.7.0) analysis tool. The measurements were taken from four different parts of the channels to ensure complete representation.

Fujifilm X-A3 camera with XC 16–50 mm objective was used to take photographs of the experiments. ImageJ image processing software was used to quantitatively determine the signals from the pictures of the metal analysis chips.

### 2.5. Final Preparations and Measuring with the Metal Analysis Chip

**Figure 1:**
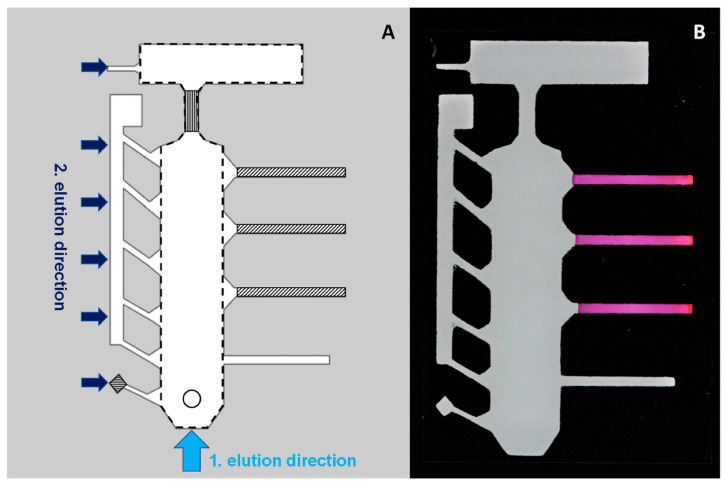
(**A**) Schematic displaying different areas and working steps of the developed metal analysis chip (on the left). The area encircled with a dashed line marks the TLC region and the smaller striped regions show different detection reagent application zones: vertical for dimethylglyoxime; horizontal for potassium ferrocyanide; diagonal for dithizone. The circle marks the sample application area and the arrows indicate the direction and order of the elution steps. (**B**) Picture of a fully prepared metal analysis chip before adding the sample.

To make printed (and pre-treated) chips ready for analysis, detection reagents were deposited to their respective zones (see Figure 1). A total of 4.5 µL of dimethylglyoxime solution was dried on the narrowed part of the thin-layer chromatography (TLC) area (vertical stripes), 1 µL of potassium ferrocyanide solution was dried on the separate small area to the bottom left corner of the chip (horizontal stripes), and 6.5 µL of 0.04% dithizone solution was dried onto each of the three channels on the right side of the chip (diagonal stripes). The fourth (bottom) channel is for balancing the eluent front flow during TLC (and later for gathering any leftover potassium ferrocyanide that might interfere with Pb detection).

To begin the analysis, 1.5 µL of the sample solution is applied and dried to the beginning of the TLC strip (0.7 mm from the edge). Firstly, the chip is eluted vertically with 0.125 M NaNO_3_ solution in the TLC chamber (lasting for approximately 13 min). After drying, the chip is rotated 90 degrees to the left and eluted again in 0.2 M sodium acetate buffer (pH = 5). The results can be detected after drying and the entire analysis could be completed in 30 min.

For quantifying the results, images of the chips were taken in a closed setup with controlled lighting conditions, keeping the camera parameters and distance from the chips constant throughout all the experiments. The images were then processed in ImageJ to determine the colour intensities for each of the metal complexes (for more details, see the Supplementary ImageJ analysis procedure). A corresponding concentration value was found based on that.

## 3. Results

### 3.1. Choice of the Binder

To create microparticle-based materials with printing, the addition of binding agents is needed. As demonstrated in our previous work [19], a common TLC mixture that uses gypsum as the binder produces poor results with SP, because the mixture will start to display unsuitable non-Newtonian (rheopectic) properties. Ideally the printing mixture for SP should exhibit shear thinning, or pseudoplastic behaviour [28]. This means that for printing silica gel microparticles, another binding agent is needed that would act both as a thickener for printing slurry and as a binder (both between the particles and to the substrate) after the material has dried. For that purpose, several food thickening agents and other common substances were tested.

After preliminary tests agarose, starch and polyethylene glycol were ruled out since they (1) did not form stable mixtures with the particles as the latter started to precipitate or (2) the mixtures were not printable, displaying similar problems as gypsum-based mixtures. These issues were not seen with sodium alginate, guar gum, and XG, and therefore those binders were chosen for further investigation. The objective was to determine which of the chosen binders would (1) have a good binding ability, even if aqueous eluents are used on the material, (2) have potentially the least impact on the particle-derived properties of the material (based on the wetting time and its variability) and (3) provide adequate printing results at the lowest concentration. In addition, a comparison with Whatman filter paper was made in terms of the wetting characteristics and material thickness. The results are presented in Table 1. For the comparison, all the binding agent solutions were made with 4 mg mL^−1^ concentration and the printing mixture was prepared using 470 mg of silica particles and 75 µL of glycerol per 1 mL of the binding agent solution.

All chosen binders yielded satisfactory or good results in terms of printing, although in case of Sigma-Aldrich XG the viscosity of the mixture was lower than with the others, which resulted in slight loss of resolution and accuracy as well as more inconsistent results. When comparing the wetting characteristics, sodium alginate proved to be an unsuitable binding agent since the material started to flake off from the glass substrate when it was submerged into water. Furthermore, for guar gum bound material the wetting took significantly longer, and the variance of the results was much higher—both between replicates and different channels in one print (see Supplementary Figure S4).

Based on these results, XG from Piprapood filled the set criteria the best, while being also one of the least expensive from the selection and it was therefore chosen for further testing. In addition, it is believed that during the drying process XG is crosslinked and bound covalently to the glass surface and silica particles, which also explains the durability of the prepared material [29].

### 3.2. Optimization of Printing Mixture and Screen Choice

**Figure 2:**
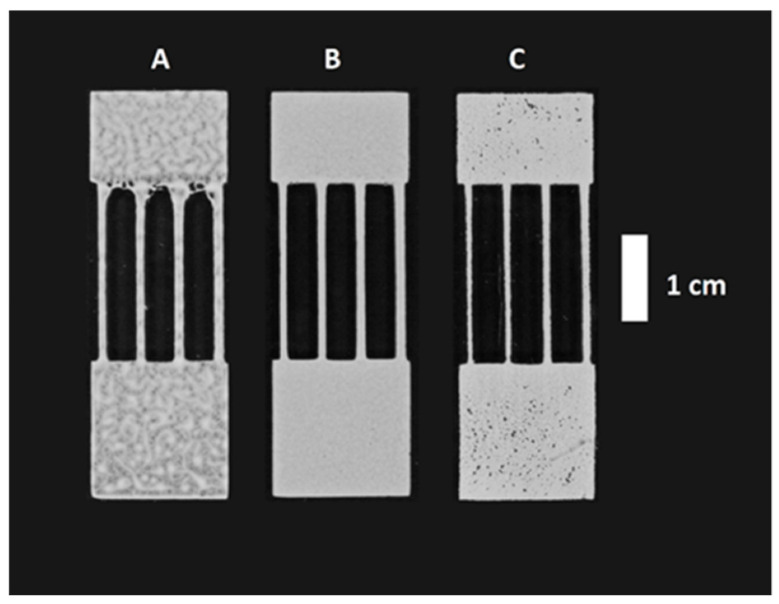
The influence of the silica gel particles concentration on the printing results: (**A**) low concentration (350 mg mL^−1^), (**B**) medium concentration (450 mg mL^−1^), (**C**) high concentration (550 mg mL^−1^). All mixtures contain 4 mg mL^−1^ XG.

To determine the optimal printing mixture composition, XG concentration was further varied from 3 to 6 mg mL^−1^ while the silica gel particles concentration varied in the range of 350 to 550 mg mL^−1^. With an XG concentration less than 3 mg mL^−1^, particles started to sediment out while at a concentration higher than 6 mg mL^−1^ clumps started forming in the binder solution. Although the mixture was usable for printing in the entire selected range, in case of lower viscosity mixtures (especially with 350 mg mL^−1^ particles) the prints had lower resolution and accuracy, more defects, and an uneven random pattern (Figure 2A). On the other hand, with mixtures containing 500 mg mL^−1^ particles or more, small “pin-head” defects started to appear, indicating uncomplete printing (Figure 2C). Therefore, the optimal range in which to operate with the used mixture components was estimated to be around 4–5 mg mL^−1^ XG with 400–500 mg mL^−1^ silica gel particles.

The optimal screen for printing is determined: (1) by the size of the used particles since the mesh hole size needs to be at least three times the particle size for uniform prints [30], and (2) by the desired resolution of the print. In this work, particles with a 15–25 µm diameter were mainly used and from the available screens, a screen with mesh openings of 142 µm proved to be the most suitable. For instance, with smaller (66 µm) mesh openings the printed shapes were inconsistent as the printing mixture did not entirely pass through the screen, while with bigger (512 µm) mesh openings a clearly distinguishable mesh pattern started to appear on the prints and the resolution and accuracy of the results was lowered (for details, see Supplementary Table S1).

The screens with higher and lower mesh densities were further used to demonstrate the possibility of varying particle size and therefore the porosity of the resulting material. For this, silica gel particles with an average diameter of 5 µm and particles in the range of 63–142 µm were used. In case of 5 µm particles, a screen with 66 µm mesh openings and for the bigger particles, a screen with 514 µm mesh openings were used (for results, see Supplementary Table S1). The difference in material porosity was observed from the material wetting times, which increased about five times for the smaller particles and was almost reduced to half with the bigger particles in comparison to the 15–25 µm silica particles (Supplementary Table S2).

### 3.3. Control over Material Thickness

The thickness of the material can be important for several reasons: in addition to determining the liquid holding capacity, it can also influence the chromatographic behaviour of the material [31]. Overall, there are multiple different ways to control the thickness of the resulting material with the current method. For instance, to increase the thickness, a screen with bigger mesh openings can be used, or the particles’ size and/or concentration in the mixture can be increased. The best choice will depend on the situation as all those approaches work in various degrees and with different limitations, e.g., changing the particles’ concentration works mostly for fine tuning, while varying their size gives more range. However, it may require a different screen as well. The influence and extent on the printed material thicknesses from various factors can be seen in Supplementary Table S2.

Moreover, an alternative approach for increasing the thickness is to print several layers consecutively on top of each other without letting the previous layer dry. As shown in Supplementary Table S2, with the addition of a second layer, the material thickness increased by 24% and with a third layer it increased another 26%. Furthermore, with a second layer the material became more homogenous and fewer small defects were apparent. On the other hand, with each additional layer the printing accuracy started to decline, increasing the channel widths on an average by 0.15 µm for two and 0.3 µm for three layers (see Supplementary Figure S5). With three layers the bigger variance was also noticeable when comparing the overall quality of the results as well as the properties of the material (wetting time and thickness). Therefore, in the authors’ opinion the best results could be reached with double print as it gives more uniform results with higher repeatability while suffering only a minimal loss of resolution.

### 3.4. Printing Accuracy

To determine the overall accuracy of the printing process (for a screen with 142 µm mesh openings), the printed channel widths were compared to their design. The comparison of the two and the channel widths in the mas, allowed us to estimate the accuracy of the cutting of the mask and the accuracy and resolution of printing itself. It is important to note that the repeatability of the cutting process as well as the mask transition process to the screen could cause further variability to the results.

From the results presented in Supplementary Figure S6 it is apparent that the accuracy of the cutting process is significantly poorer than 0.1 mm and the resulting channels in the mask and consequently in the printed form are larger than the ones designed. However, when comparing the channels in the mask with the printed results then they match better than the design with the mask matches, which shows reasonable accuracy for printing itself. These results also indicate that more accurate results could be achieved with better cutting equipment. In terms of the currently used equipment, the narrowest achievable channel width (i.e., resolution) is approximately 0.5 mm and the overall accuracy of SP can be estimated to be at or below 0.2 mm.

### 3.5. Development of the Metal Analysis Chip

To demonstrate the potential of using SP for the creation of a functional particle-based microfluidic device, a chip was developed that allows for the detection of Cd (II), Cu (II), Pb (II), Ni (II), and Fe (III) from a mixed sample. To highlight the rapidness and simplicity of prototyping with the SP method, a quick revision and time estimate is given on the steps for producing the chip. With the design ready, it takes approximately 1 h to cut the mask, apply it on the screen, and print a testing batch of 10 replicates with it. This is followed by a 2-h drying process (which might be shortened by increasing the temperature) and the interference pre-treatment, which takes about 1 h per chip. The latter, however, is only necessary because of the currently used particles and detecting reagents and could be disregarded with a different setup. Finally, the detection reagents are added, and the chip is ready to use, taking approximately 4 h from design to testing.

**Figure 3:**
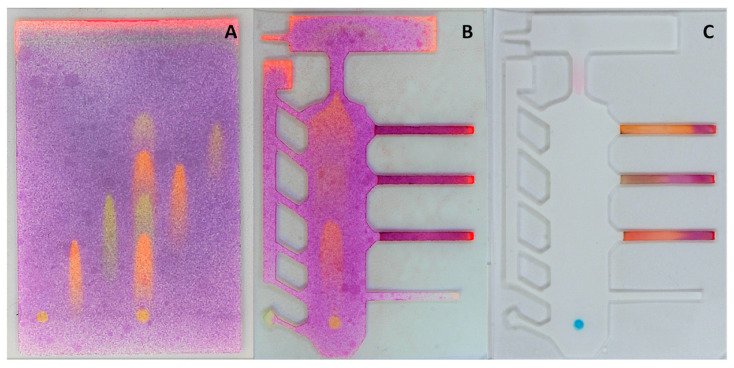
(**A**) Separation of metals on a printed TLC plate using 0.125 M NaNO_3_ solution (from left to right: Fe, Pb, and Cu mixture of the five, Cd, Ni). (**B**) Separation of the mixture on the developed chip. The plates are sprayed with 0.02% dithizone solution. (**C**) Results after the full two-step process. In all cases the metals concentration was 1 mM and the added sample amount for the plain TLC was 1 µL and 1.5 µL for the designed chips.

The goal for the microfluidic chip was to separate analytes in one direction using TLC and then use a second elution step (perpendicular to the first) to complete the detection (for more details see Figure 1). For this, the movement and separation of all the chosen metals was first investigated on a plain printed TLC plate (Figure 3A). The applied separation principle could be considered close to ion-exchange chromatography, where the silica surface acts as a cation exchanger [32]. Among the metal ions, Ni had the highest mobility, followed by Cd, Cu, and Pb, respectively, while Fe was immobile with the used eluent. Despite separating all the analytes, significant tailing was present for metals that moved, and the final separation was poor. However, considerable improvement was achieved with a specially designed chip at the same elution conditions (Figure 3B). Although tailing of the metal spots still occurred, the analytes were more clearly separated from each other. This can be attributed to lowering the eluent flow rate that leads to less tailing due to mass transfer effects [33]. The flow rate is lowered by a narrower initial contact area with the eluent, small channels to both sides of the TLC region and the widening in the top [34,35]. In addition to slowing down the eluent flow, the latter and the side channels also increase the relative length of the TLC area. Overall, this also demonstrates the potential of using SP for creating customized TLC plates to improve their separation and performance, especially as the thickness of the material can be controlled simultaneously.

Three alternative approaches for detection with different reagents were applied on the chip. Nickel ions are caught when passing through the bottleneck during the TLC step and the formed pink complex is nearly immobile in the eluent flow. Therefore, the visualization of the analyte takes place already during the TLC phase. For Cd, Cu, and Pb, the metals are directed from the TLC area to the dithizone covered detection strips during the second elution step. This helps to compensate the spreading and tailing of the analytes, since most of the “tail” is also collected to the detection zone. Finally, for Fe (III) detection, potassium ferrocyanide is guided over the sample addition area during the second elution step and an immobile blue complex is formed. An example of the result for 1 mM mixture of the analytes can be seen in Figure 3C.

### 3.6. Calibration Measurements

**Figure 4:**
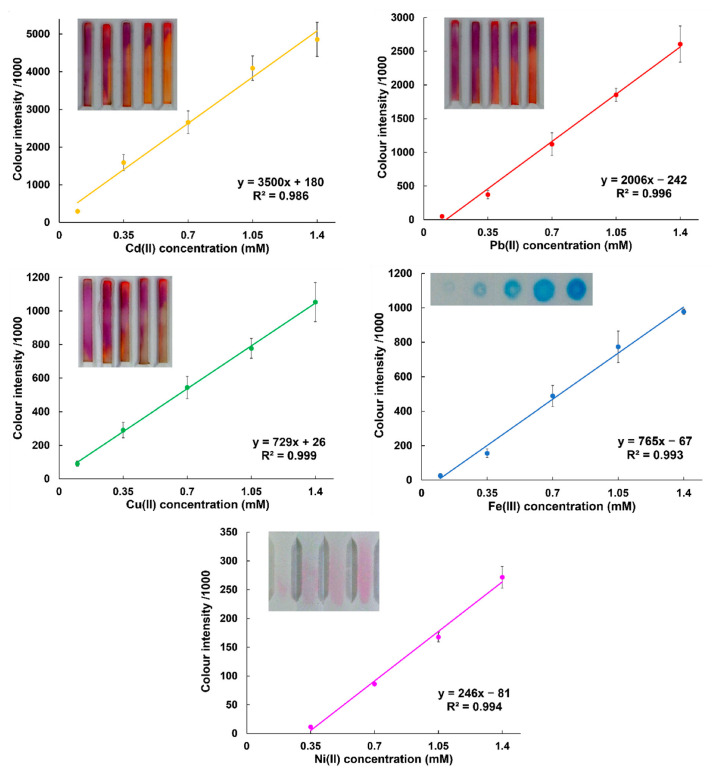
Calibration curves with the representative images of the signals for the detection of Cd (II), Pb (II), Cu (II), Fe (III), and Ni (II) on the printed microfluidics chips (n = 4). The determined intensity values were divided by 1000.

Based on the colour intensities (determined with ImageJ from images of the chips), linear dynamic range, detection limit, and chip reproducibility were investigated. For that mixed calibration solutions were used, where each metal concentration was separately varied in the range 0.1–1.4 mM while keeping the total analyte concentration at 3.6 mM. This allowed us to use only five calibration solutions and consider any risk of interference between the analytes themselves. An example of colour intensities and linear calibration curves for each of the analyte metals is shown in Figure 4. The linear range for Cu, Cd, Pb, and Fe can be estimated to be between 0.2 and 1.4 mM while for Ni it was between 0.3 and 1.4 mM. The upper limit of linear range could be increased for all the analytes with the current setup by further measurements except for Cd, in which case it already started to go slightly beyond linearity. Regarding the detection limit, for Cu, Cd, and Pb it could be estimated to be around 0.1 mM, as this was still clearly visually confirmable. For Fe, the same applied if the solution was freshly prepared, since only the free ionic form of Fe (III) can be detected. Finally, for the detection of Ni, the estimated limit of detection was 0.14 mM. The relative standard deviation in the middle of the calibration curve varied between 10 and 15%.

An important factor to consider in terms of variability of the results as well as the relatively high limit of detection is the low sample amount (1.5 µL) and that slight fluctuations in adding the sample could have a considerable effect on the results. Both of these issues could be solved by increasing the sample amount up to 50 or 150 µL by integrating a printed solid-phase extraction area in front of the TLC. Regarding the current setup, silica gel particles with a strong cation exchange surface (e.g., with propylsulphonic acid groups) could be used. Moreover, to improve the detection limit, more sensitive methods could be applied, e.g., fluorescence detection, if suitable reagents and quantification devices are available. On the other hand, the upper limit of linear range could easily be further improved (at least for all metals except for Fe) by increasing the concentration of the detecting reagent or the area of the detection zone.

### 3.7. Interference Studies

Since the calibration measurements were conducted with varying mixtures of all the analyte metals, it demonstrates the lack of their own relative interference in similar concentrations (up to 10 times difference). However, it must be noted that in case of detecting with dithizone, it was not strictly necessary to separate the analytes (e.g., Cd and Cu) into different detection channels. Depending on their relative concentration, a mix of two metals also appeared sometimes in one detection channel (see Supplementary Figure S7). However, due to the clearly distinguishable colours and differences in complex strength with dithizone, they formed isolated areas that could be separated by masking in ImageJ. The similar effect might also realize in case of other metals that form complexes with dithizone (e.g., Zn or Mn) [36]. Therefore, interference between metals does not occur unless both the retention factor during TLC and the colour of the complex are similar.

**Figure 5:**
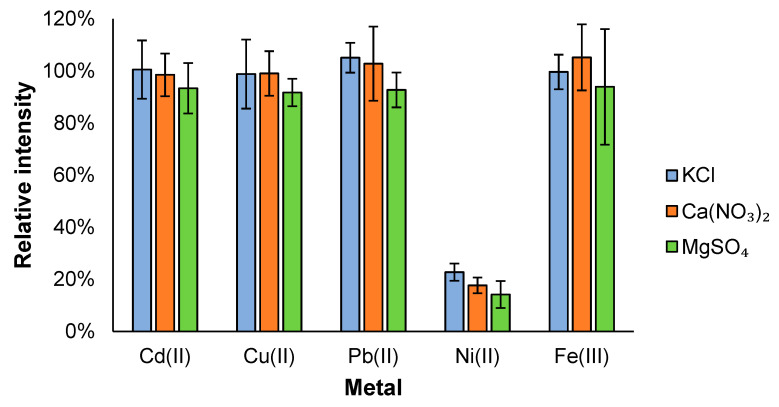
An interference study with a 0.7 mM analyte mixture in a 0.1 M background salt solution. Differently coloured columns correspond to different background ions. The measured intensities (n = 4) are compared against (divided by) intensity values determined from the calibration measurements.

To investigate the effect of other potentially interfering ions found in environmental samples that are also present in much higher concentrations, solutions containing each of the analyte metals at 0.7 mM with the background of 0.1 M KCl, Ca(NO_3_)_2_, and MgSO_4_ were prepared. This resulted in 143 times excess of those background ions. Moreover, only freshly mixed samples were used to avoid any unwanted precipitate formation. The related data are presented in Supplementary Table S3 and comparative results to the calibration measurements are shown in Figure 5. Overall, the change in intensity remained around 10% for Cd, Cu, Pb, and Fe, which matches the uncertainty of the calibration results, themselves suggesting an insignificant effect from these interfering ions. However, with MgSO_4_ there was a small drop in intensity for all metals and in case of Fe, a fast drop in signal intensity between the replicates in the same measurement session was observed.

In case of Ni, the influence of the high salt background was very significant with up to a 10-fold drop in intensity. This is mostly attributed to the higher mobility of Ni. By having a high salt content in the sample, the Ni ions move completely in the eluent front and are spread out to the side channels and edges of the chip, decreasing the detected amount. This could either be solved by redesigning the chip or simply avoiding samples with a high overall ionic content for Ni detection.

**Figure 6:**
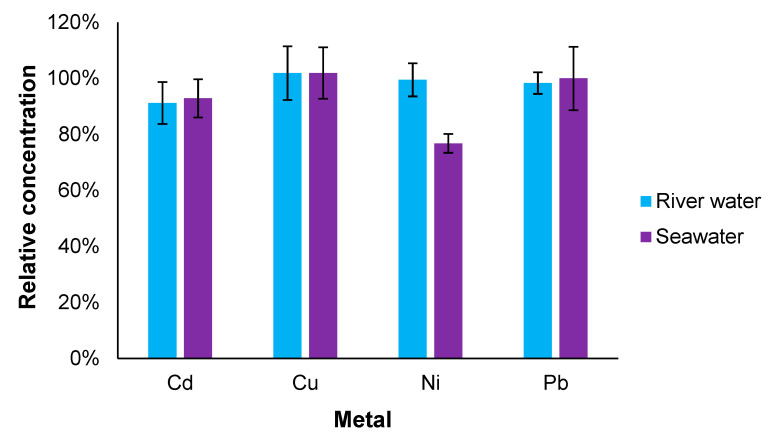
Sample matrix study with spiked river and seawater samples. Values from metal analysis chips (n = 3) are compared against the determined results with MP-AES (i.e., values determined with metal analysis chips are divided by MP-AES measurement results). For the determined values, see Supplementary Table S4.

To investigate any further interferences for the developed chip, spiked environmental samples were created with river and seawater (with the analyte ions concentration being kept relatively in the middle of the linear range). The river samples were obtained from Pärnu River (Pärnu, Estonia) and seawater samples were collected from coastal seawater of the Baltic Sea. The obtained results were compared to microwave plasma-atomic emission spectrometer (MP-AES) analysis of the same samples (Figure 6). Overall, most of the results are in good correlation, with a small difference for Ni in case of seawater samples. However, this was expected as it had already been observed in the previous interference measurements with high background salinity. Moreover, in case of the river samples, the difference with the standard method was negligible, showing better feasibility for measuring Ni in real samples.

The results concerning Fe are omitted from Figure 6 as no reliable values were obtained with the metal analysis chip. The first reason being the formation of precipitate that was seen already during spiking of the samples, which significantly lowered the free ionic form of Fe(III). However, the second issue was a background signal coming from the solution (most likely due to the formed precipitate). This meant that although some colour change was still visible, the previously applied quantification algorithm did not work anymore. On the other hand, it highlights that the detection approach used for other analytes is more preferrable, as they are separated from the initial sample matrix, avoiding similar problems. Therefore, the overall performance of the chip could be regarded as a success since the separation performed as expected and no other significant interferences did occur.

## 4. Conclusions and Future Perspectives

In the current work, SP was used to produce layered particle-based materials with a controllable shape to form microfluidics systems. XG worked as an excellent choice for printing and binding particles together (and to the substrate) while displaying the ability to hold the material intact even after long and repeated exposure to aqueous solvents. Several printing parameters (screen mesh, particle size and concentration, etc.) were varied and their impact on the resulting material (thickness, wetting characteristics, resolution) was investigated. In this regard, the purpose of the current work was to demonstrate the potential of SP and give general guidelines for producing similar particle-based materials with controlled shapes. Furthermore, the speed and simplicity of the process as well as the low cost and environmental friendliness are a significant advantage of the approach.

In terms of the exemplary developed analysis chip, several novel solutions were demonstrated. Among them, using TLC to create a multi-analysis microfluidic test and altering the shape of the material to improve its separation of species could be considered most important. The performance of the chip was also successful, and it displayed robustness concerning potential interferants present in the samples. Despite the detection limits currently being unsuitable for most real-world applications, there is still considerable room for improvement through increasing sample amounts. This, combined with integrated eluent reservoirs and a portable quantifying device could also make these tests suitable for various other chemical screening applications, including monitoring minerals in drinking water, nutrients, and pesticides in agriculture and food industry, as well as determining a variety of compounds (minerals, vitamins, therapeutic drugs, etc.) in point-of-care testing.

## Figures and Tables

**Table 1:** Comparison of the different binding agents ^a^.

	Sodium Alginate	Guar Gum	XG (Piprapood)	XG (Sigma)
Ionic or neutral in water	Ionic	Neutral	Ionic	Ionic
Mixture printability	Good	Good	Good	Satisfactory
Material withstands water	No	Yes	Yes	Yes
Material wetting time (s) ^b^	-	83.9 ± 7.0	52.6 ± 2.2	54.4 ± 2.5
Thickness of the printed material (µm) ^c^	112.6 ± 9.1	120.2 ± 8.4	109.4 ± 4.5	110 ± 11

^a^ The corresponding wetting time for Whatman filter paper was 197 ± 27 s and the measured thickness of the paper was 182.9 (3.7) micrometres. ^b^ average and standard deviation from 15 replicates. ^c^ average and standard deviation from 18 replicates.

## Data Availability

The data presented in this study are available either in the Supplementary Material or can be made available on request.

## Supplementary Materials

The following supporting information can be downloaded at: https://www.mdpi.com/article/10.3390/mi14071369/s1, Figure S1: Interference with dithizone. Figure S2: Printed shapes for optimization. Figure S3: Illustration of the SP process. Figure S4: Wetting time measurements. Figure S5: Channel widths with multiple layers. Figure S6: Accuracy of printing method. Figure S7: Separating mixed analyte signals. Table S1: Examples with different screens and particles. Table S2: Determined thickness and wetting values. Table S3: Interference measurements. Table S4: Spiked sample measurements.
