# Supplementary material for: Screen Printed Particle-Based Microfluidics: Optimization and Exemplary Application for Heavy Metals Analysis
Source: Micromachines (Basel). 2023 Jul 4;14(7):1369. doi: 10.3390/mi14071369 (PMC10386728; doi:10.3390/mi14071369)
Supplement: Supplementary file 1 [file micromachines-14-01369-s001.zip › micromachines-2482286-supplementary.pdf]

Supplementary Materials

Screen Printed Particle-Based Microfluidics: Optimization and Exemplary Application for Heavy Metals Analysis

Indrek Saar * and Hanno Evard

Institute of Chemistry, University of Tartu, Ravila 14a, 50411 Tartu, Estonia;
*E-mail: indrek.saar@ut.ee

Overview of the included data

Image J analysis procedure

Interference with dithizone

Figure S1 – Interference with dithizone

Figures and tables

Figure S2 – Printed shapes for optimization

Figure S3 – Illustration of the SP process

Figure S4 – Wetting time measurements

Figure S5 – Channel widths with multiple layers

Figure S6 – Accuracy of printing method

Figure S7 – Separating mixed analyte signals

Table S1 – Examples with different screens and particles

Table S2 – Determined thickness and wetting values

Table S3 – Interference measurements

Table S4 – Spiked sample measurements

Image J analysis procedure

The analysis algorithm varied slightly depending on the analyte metal, but the general principles remained same:

1. The desired detection area was cropped out.
2. Image was split into red, green and blue colour channels.
3. Based on the strongest contrast between the two channels (or their inverted forms) one or several subtraction operations and their following combination was conducted. (e.g. for Fe inverted blue channel was subtracted from the inverted red and green channels and these results were then merged.)
4. Based on the result, automatic thresholding algorithm was used to create a mask limiting the collected signal area.
5. Finally, the previous two steps were combined to calculate the sum of pixel intensities over the limited area (see Figure S7 for the limited intensity areas of Cd and Cu).
6. The collected data was used for further analysis in Microsoft Excel.

Interference with dithizone

Performing only TLC step, no interference was present with using LiChroprep particles. However, in case of two-step elution, interfering compounds present in the printed material were concentrated over the dithizone detection region, giving strongly coloured (pink/red) signal that interfered with the detection of analytes. This led to testing different silica gels from other manufacturers among which Davasil grade 710 particles appeared to be most suitable. Although the interference was still present (Figure S1 B and C), it could be more easily eliminated by pre-treatment steps. For this the printed chip was eluted through with 0.1 M $NaNO_3$ solution followed by deionized water, which moved those compounds (and $NaNO_3$) to the edges of the design, where their presence would not disturb further analysis. This approach was deemed less invasive and more time-efficient than developing necessary protocol for treating the particles before printing. Moreover, several aspects of the final design, such as the inclined channels on the left side of the chip and the square area on top of them were added to minimize eluting the interfering compounds back out of the corners and edges they had previously been concentrated to (see Figure 3B).

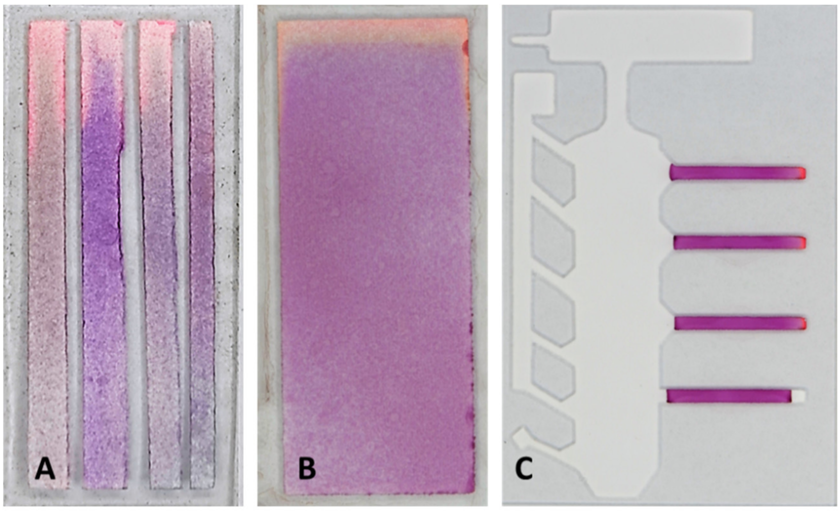

Figure S1. Interference (visible as the pink eluted front) occurs when using dithizone as detecting reagent. (**A**) Printed material strips with LiChroprep particles and eluted with different eluents. (**B**) Printed material with Davisil particles and eluted with 0.1 M $NaNO_3$ and DI water. (**C**) Printed metal analysis chip with Davisil particles after pre-treatment steps, concentrated interferants visible at the end of detection channels.

Several experiments were also conducted to determine the cause for the interference. It was hypothesized that some other metal was present in the silica gel, which forms a complex with dithizone. However, after elemental analysis using scanning electron microscopy energy dispersive spectroscopy and laser ablation inductively coupled plasma mass spectrometry analysis and spot tests with several potential metals and different detecting reagents, the results remained inconclusive. Since a more thorough investigation was deemed out of scope for the current paper, the approach to pre-treat the formed material was deemed to be sufficient in terms of the set goals. For future reference either a more thorough investigation of different silica gel particles or an efficient purifying process prior to printing the particles into a chip could be conducted to avoid these steps.

Figures and tables

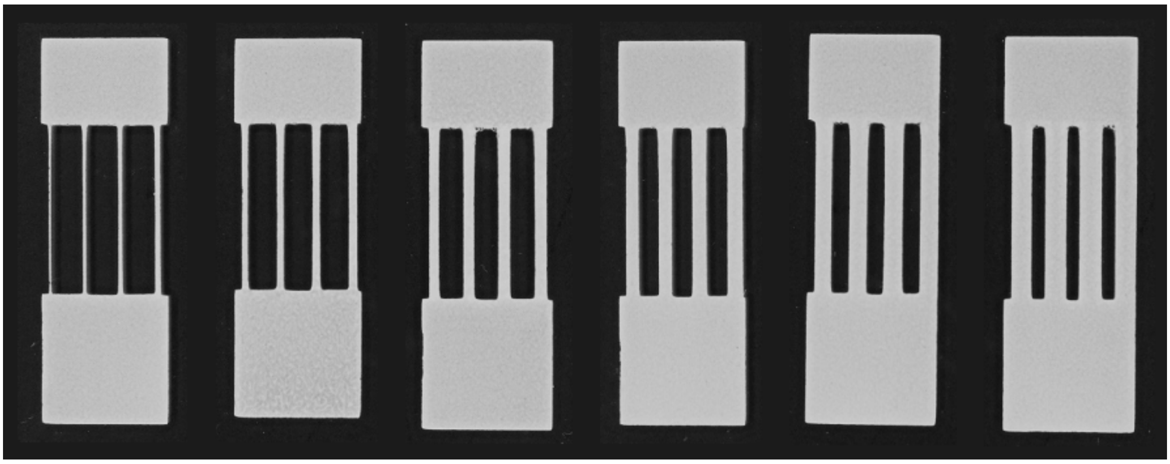

Figure S2. Six printed shapes for optimization, designed channel width varies from 0.1 mm to 2.4 mm.

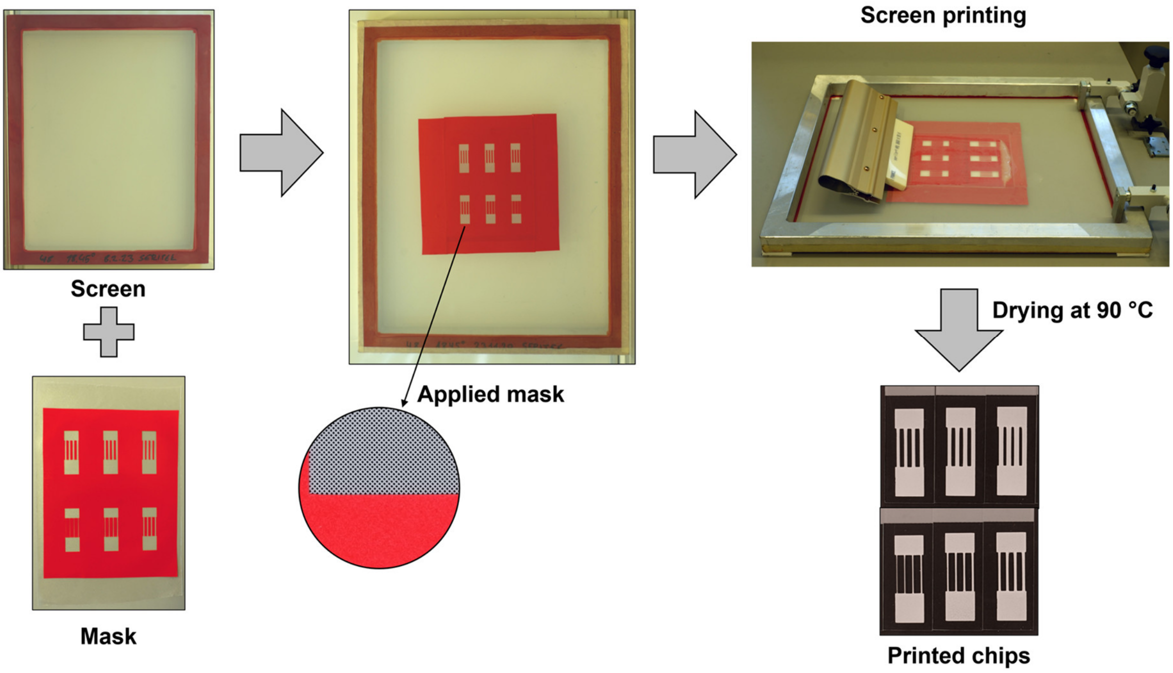

Figure S3. Illustration of the SP process: a mask is cut out of removable vinyl and applied to the screen with the help of a transfer film. The screen is fixed into setup and the printing mixture is applied on the screen. During printing, the mixture is forced through the screen on to the substrate(s) with squeegee resulting in printed chips. Finally, the chips are dried in the oven at 90 degrees.

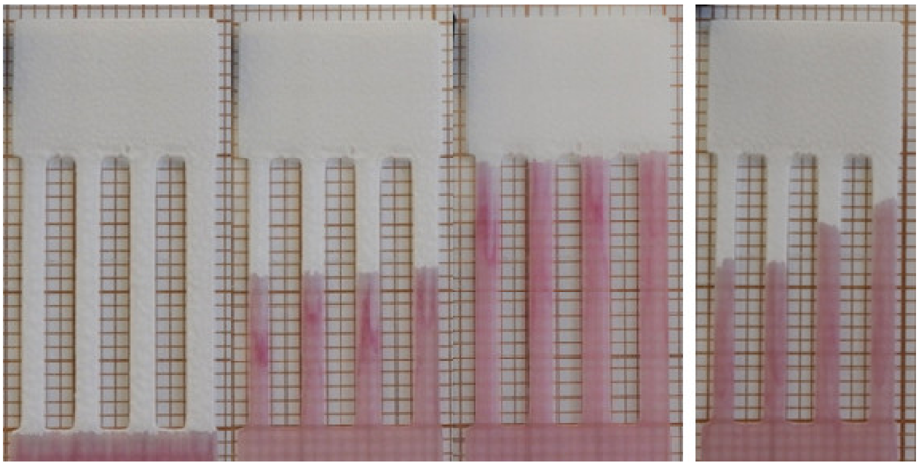

Figure S4. Examples of wetting time measurements: material with XG as the binder (three leftmost images) demonstrates even wetting throughout the material, while guar gum as the binder (rightmost image) shows considerable discrepancies. It must be noted that the wetting time also depends on the geometry of the material – going from larger area to narrower channels will result in slightly increased wetting speed and hence moving from wider channels to narrower this effect is increased [1,2]. This is also an important contributor to the standard deviation of the wetting time estimates when data is used from channels with different widths. Furthermore, in case of the narrowest channels even small defects start to significantly influence the results. Therefore, to get more realistic estimation on the material own wetting characteristics, only shapes with channel widths of 0.5 mm and bigger are included in data analysis.

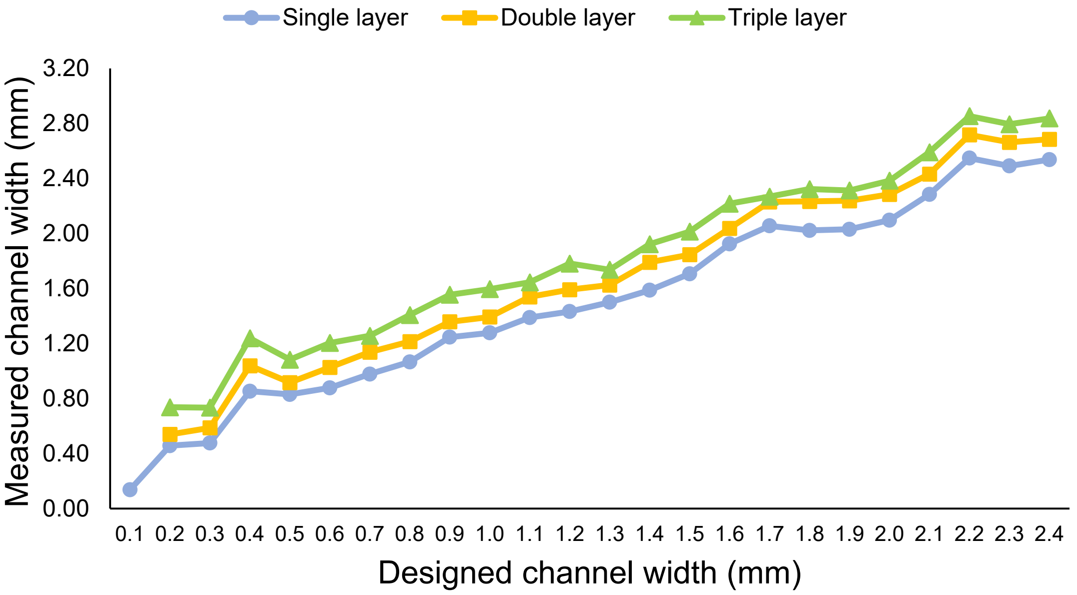

Figure S5. Comparison of channel widths for multiple printed layers. In case of the channel with 0.1 mm designed width, not enough reliable results were obtained for double and triple layers.

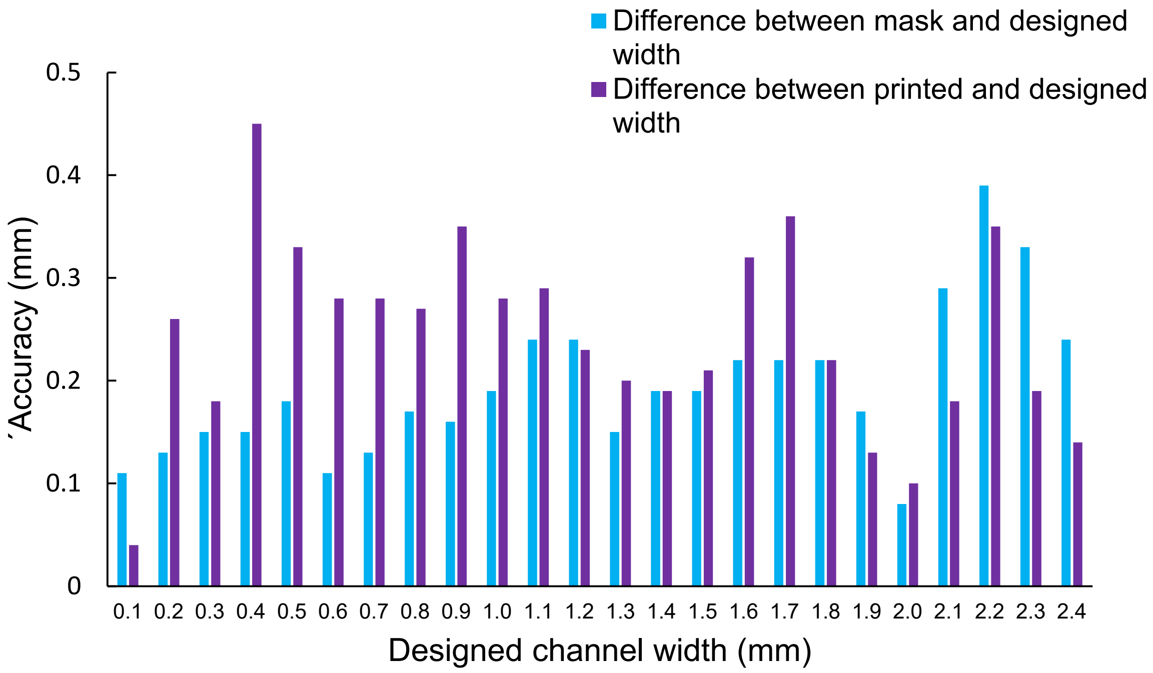

Figure S6. Printing accuracy of the channels. The printed channels are systematically wider compared to the designed width, however the main component for this deviation is the mask width as the printed channels differ less and more randomly from it. Based on data, approximately 0.5 mm can be considered as the narrowest achievable channel width. This was concluded from repeatedly achieving intact channels for designed width of 0.2 and 0.3 mm while the narrowest channel (0.1 mm designed width) was often fragmentary. In case of designed channel width of 0.4 mm, there was an issue with vinyl transfer to the screen, which resulted in abnormally high printed channel widths for that channel.

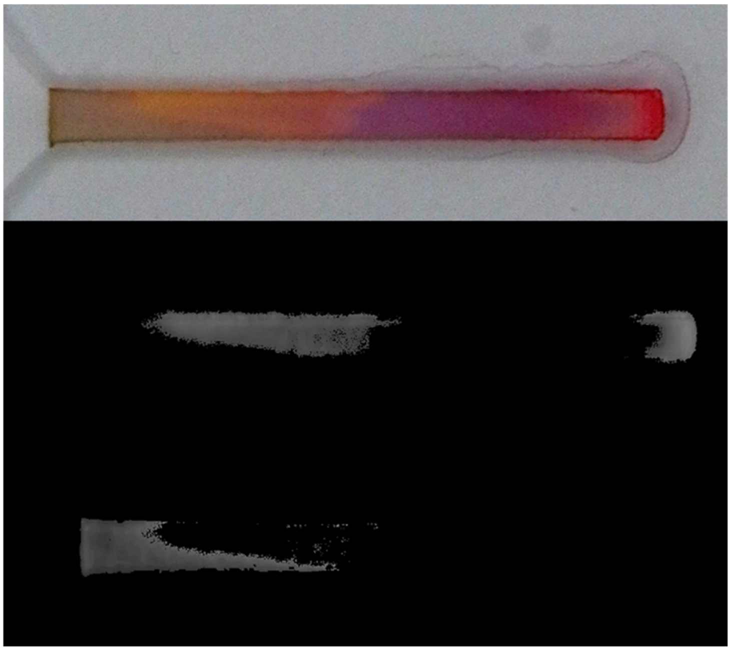

Figure S7. Example of a mixed signal (Cu and Cd) in one detection channel and the separation of (their respective) signals with masking (in ImageJ with automatic thresholding).

Table S1. Examples of printed materials from screen and particles testing.

<table>
<tr><td colspan="2">Screen mesh openings: 63 μm</td><td>Screen mesh openings: 142 μm</td><td colspan="2">Screen mesh openings: 514 μm</td></tr>
<tr><td>Particles size: 5 μm</td><td colspan="3">Particles size: 15-25 μm</td><td>Particles size: 63-142 μm</td></tr>
<tr><td>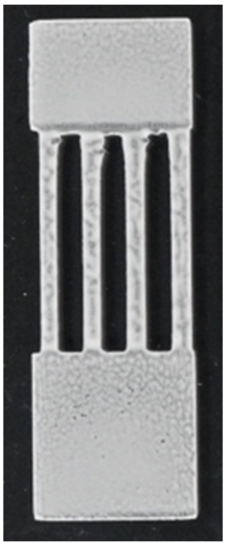</td><td>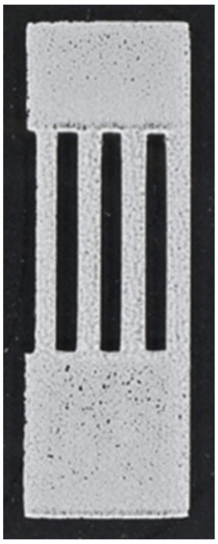</td><td>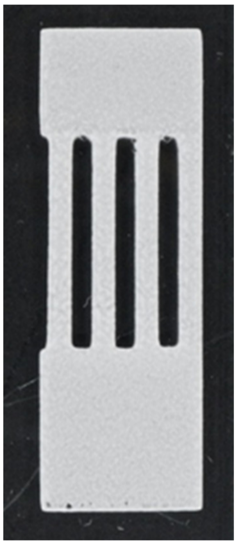</td><td>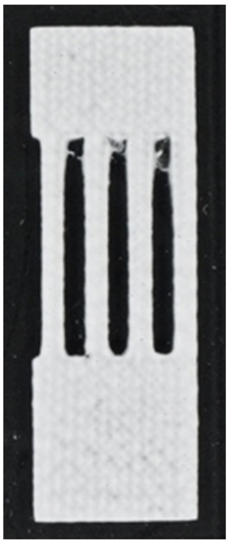</td><td>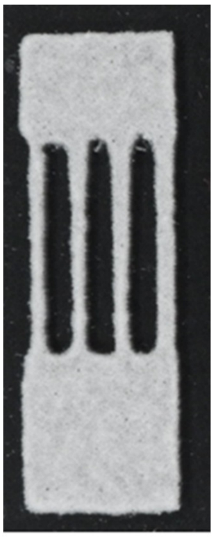</td></tr>
</table>

Table S2. Control over material thickness and wetting time.

**Adjusted parameter**	**Average thickness**	**Relative thickness**	**Wetting time**
Optimal mixture and conditions (470 mg mL^{-1} particles)	109.4 ± 4.5	100 %	52.6 ± 2.2 s
Mixture with 350 mg mL^{-1} particles	86.1 ± 6.6	78.7 %	47.2 ± 3.7 s
Mixture with 550 mg mL^{-1} particles	172.7 ± 9.3	158 %	41.5 ± 1.3 s
Smaller (63 μm mesh) screen	106.1 ± 9.5	97.0 %	75.4 ± 8.1 s
Bigger (514 μm mesh) screen	210.7 ± 8.8	193 %	54.4 ± 3.8 s
Smaller (5 μm) particles	63.4 ± 9.8	58.0 %	266 ± 36 s
Bigger (63-142 μm) particles	346 ± 14	316 %	28.7 ± 2.8 s
Double printed layer	135.4 ± 6.8	124 %	48.8 ± 2.7 s
Triple printed layer	164 ± 20	150 %	52.5 ± 4.1 s

Uncertainty of the measurements are calculated using standard deviation of the replicates. For wetting time results n = 5, for thickness n = 6.

Table S3. Interference measurements with 0.1 M KCl, $Ca(NO_3)_2$ and $MgSO_4$ background solutions.

	KCl		$CaNO_3$		$MgSO_4$	
**Metal**	**Intensity**	**RSD**	**Intensity**	**RSD**	**Intensity**	**RSD**
Cd (II)	2675029	11%	2620873	8%	2484117	10%
Cu (II)	537379	13%	538709	9%	498720	6%
Pb (II)	1179699	5%	1153973	14%	1040751	7%
Ni (II)	19672	15%	15283	17%	12261	36%
Fe (III)	486361	7%	513656	12%	458571	24%

Intensity values and relative standard deviations are based on measured signals with metal analysis chip (n = 4).

Table S4. Determined metal concentrations (mM) from spiked samples.

Analysis	Sample	Fe	Cd	Cu	Ni	Pb
MP-AES	River water	0.761 ± 0.006	0.772 ± 0.003	0.741 ± 0.004	0.726 ± 0.005	0.701 ± 0.007
	Seawater	0.756 ± 0.007	0.736 ± 0.003	0.772 ± 0.004	0.739 ± 0.001	0.743 ± 0.007
Metal chip	River water	NaN	0.70 ± 0.05	0.75 ± 0.07	0.72 ± 0.04	0.69 ± 0.03
	Seawater	NaN	0.68 ± 0.05	0.79 ± 0.07	0.57 ± 0.02	0.74 ± 0.08

Uncertainties of the values are presented with standard deviation. In case of the values measured with metal analysis chip n = 3.

References

1. Liu, M.; Suo, S.; Wu, J.; Gan, Y.; AH Hanaor, D.; Chen, C.Q. Tailoring Porous Media for Controllable Capillary Flow. *J. Colloid Interface Sci.* **2019**, *539*, 379–387, doi:10.1016/j.jcis.2018.12.068.
2. Fu, E.; Ramsey, S.A.; Kauffman, P.; Lutz, B.; Yager, P. Transport in Two-Dimensional Paper Networks. *Microfluid. Nanofluidics* **2011**, *10*, 29–35, doi:10.1007/s10404-010-0643-y.
